# Effect of inoculated and uninoculated aeration pretreatment on nutrients and phytotoxicity of anaerobic digestion effluent

**DOI:** 10.1038/s41598-018-32141-7

**Published:** 2018-09-17

**Authors:** Bangxi Zhang, Yun Li, Shuyan Li, Guoxue Li, Qiaoping Sun

**Affiliations:** 1 Beijing Key Laboratory of Farmland Soil Pollution Prevention and Remediation, College of Resources and Environmental Sciences, China Agricultural University, Beijing, 100193 China; 2 Institute of Agricultural Resources and Environment, Guizhou Academy of Agricultural Sciences, Guiyang, 550006 China

## Abstract

This study investigates the influence of inoculated and uninoculated aeration pretreatment on nutrients and phytotoxicity of anaerobic digestion (AD) effluent. Swine manure AD effluent was inoculated with activated and anaerobic sludge, respectively. Our results show that aeration with the addition of activated sludge could reduce the phytotoxicity of AD effluent. Compared to the control treatment without any sludge addition, the addition of activated sludge resulted in a more significant reduction in the AD effluent salinity, NH_4_^+^ content, and organic matter (indicated by the chemical oxygen demand) when AD effluent was aerated for less than 8 hours. As a result, a much higher seed germination index (GI) was observed for the treatment with activated sludge addition, particularly when aerated for 4–5 hours, contributing the gas/water ratio of 20:1–30:1. On the other hand, no significant differences in the nutrient contents and GI value were observed for the control treatment and that with the addition of anaerobic sludge. Results from this study shed light on optimizing the management of AD effluent for agricultural application.

## Introduction

Meat consumption increases rapidly, particularly in developing countries, resulting in the emergence of large-scale, industrial livestock farms and associated facilities, such as anaerobic digestion plants, for the treatment of livestock feces^1–4^. Anaerobic digestion (AD) of livestock feces and other organic residues can be used to produce both renewable energy and organic fertilizer, in the form of biogas and digestate, respectively^5,6^. The fertilizer value of digestate depends on the presence of nutrients in the feedstock, digestion processes and subsequent storage conditions. Nevertheless, digestate can be a very useful organic fertilizer to offset the financial as well as the environmental costs associated with mineral and chemical fertilizers^7^.

Digestate can be separated into liquid and solid fractions for better management. The liquid fraction (i.e., AD effluent) and solids of digestate need to be stored and handled separated. It is recommended that the higher dry matter and fibrous fraction should be stored without disturbance, or even composted, in order to avoid any methane emission. On the other hand, AD effluent can be stored and then used as liquid fertilizers in agricultural applications. Nevertheless, macromolecular organic substances in livestock feces can be biologically decomposed into small molecule organic acids during AD process. The residual of these organic acids in AD effluent may cause phytotoxicity to crops. Thus, the pretreatment of digestate, for example, by dilution and aeration, is necessary before using as organic fertilizers for crops.

Aeration plays an important role for the biological degradation of organic matter in wastewater treatment^8–10^. Thus, aeration pretreatment can be potentially applied to condition the AD effluent by providing aerobic conditions for the oxidation of unfavorable organic acids^11,12^. Moreover, aeration can lead to ammonium (NH_4_^+^) stripping to reduce subsequent wastewater treatment load^13–15^. Nevertheless, to date, no studies have been conducted to investigate the effects of aeration pretreatment on the phytotoxicity of AD effluent, particularly with sludge addition for process optimization.

This study aims to investigate effect of inoculated and uninoculated aeration pretreatment on nutrients and phytotoxicity of anaerobic digestion effluent. Both activated and anaerobic sludge were inoculated to swine manure AD effluent. The nutrient contents and phytotoxicity of AD effluent after aeration were determined. Results from this study provide important insights to AD effluent pretreatment for agricultural application.

## Results and Discussion

### Physicochemical characteristics of AD effluent

Key physicochemical characteristics of AD effluent (i.e., digestate supernatant) were shown in Table 1. The AD effluent contained major fraction of dissolved organic substances in digestate, as indicated by COD measurement. Total phosphorus (TP) in the AD effluent was relatively low due to its residuals in the solid fraction of digestate^16^. Similar profiles were also observed for some dominant heavy metals, including Cu, Fe, and Zn. A relatively low concentration (less than 0.6 mg/L) of Cd, Cr, and Pb were measured in AD effluent. Moreover, AD effluent contained approximately 1539 mg/L amino acids, which is a valuable fertilizer for plants and crops^17,18^.

**Table 1:** Physicochemical characteristics of AD effluent (mg/L).

Parameters	AD effluent
pH	7.7 ± 0.1
COD	1468.9 ± 58.9
TN	868.9 ± 23.6
TP	29.4 ± 1.1
TK	406 ± 12.4
Cu	0.2 ± 0.1
Mg	59.3 ± 4.3
Fe	1.0 ± 0.1
Zn	2.0 ± 0.8
Total amino acids	1539.6 ± 53.6

### Variation of pH and electrical conductivity

**Figure 1:**
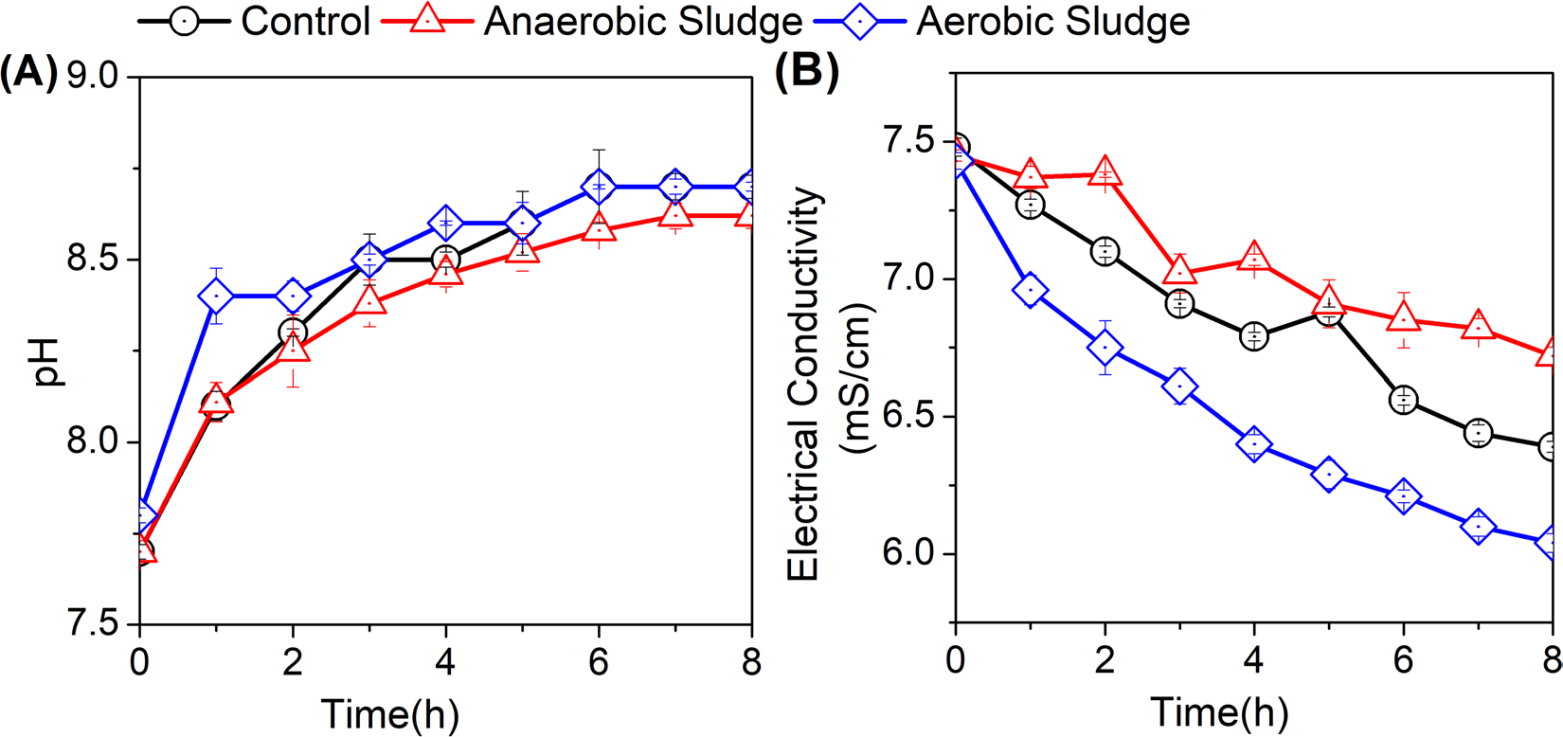
Effects of aeration on AD effluent pH (**A**) and electrical conductivity (**B**) with sludge addition. Activated and anaerobic sludge were added respectively to obtain the MLSS of 200 mg/L. AD effluent without sludge addition was set as a control. Aeration rate was maintained at 0.036 m^3^/h for all treatments.

Effects of aeration on AD effluent pH and electrical conductivity (EC) with sludge addition were shown in Fig. 1. As noted from Fig. 1A, AD effluent pH increased continuously with aeration for all treatments. This observation was caused by the decomposition and volatilization of organic acids^19^. Moreover, aeration could lead to the dissolution and release of carbon dioxide and thus an increase in AD effluent pH^20^. Of the three treatments in this study, adding aerobic and anaerobic sludge resulted in the highest and lowest increase in AD effluent pH, respectively. This result was possibly due to the improved animation of NH_4_^+^ with the addition of activated sludge, enhancing the hydroxide (OH^−^) concentration in the AD effluent for higher pH increase.

EC can simply indicate the salinity of liquid fertilizer and its suitability for agricultural applications^21^. As shown in Fig. 1B, EC decreased with aeration for all treatments, possibly owing to the precipitation of inorganic salts with pH increase (Fig. 1A). Indeed, the addition of activated sludge led to the most significant decrease in the AD effluent salinity.

### Variation of Amino acids and organic matter

**Figure 2:**
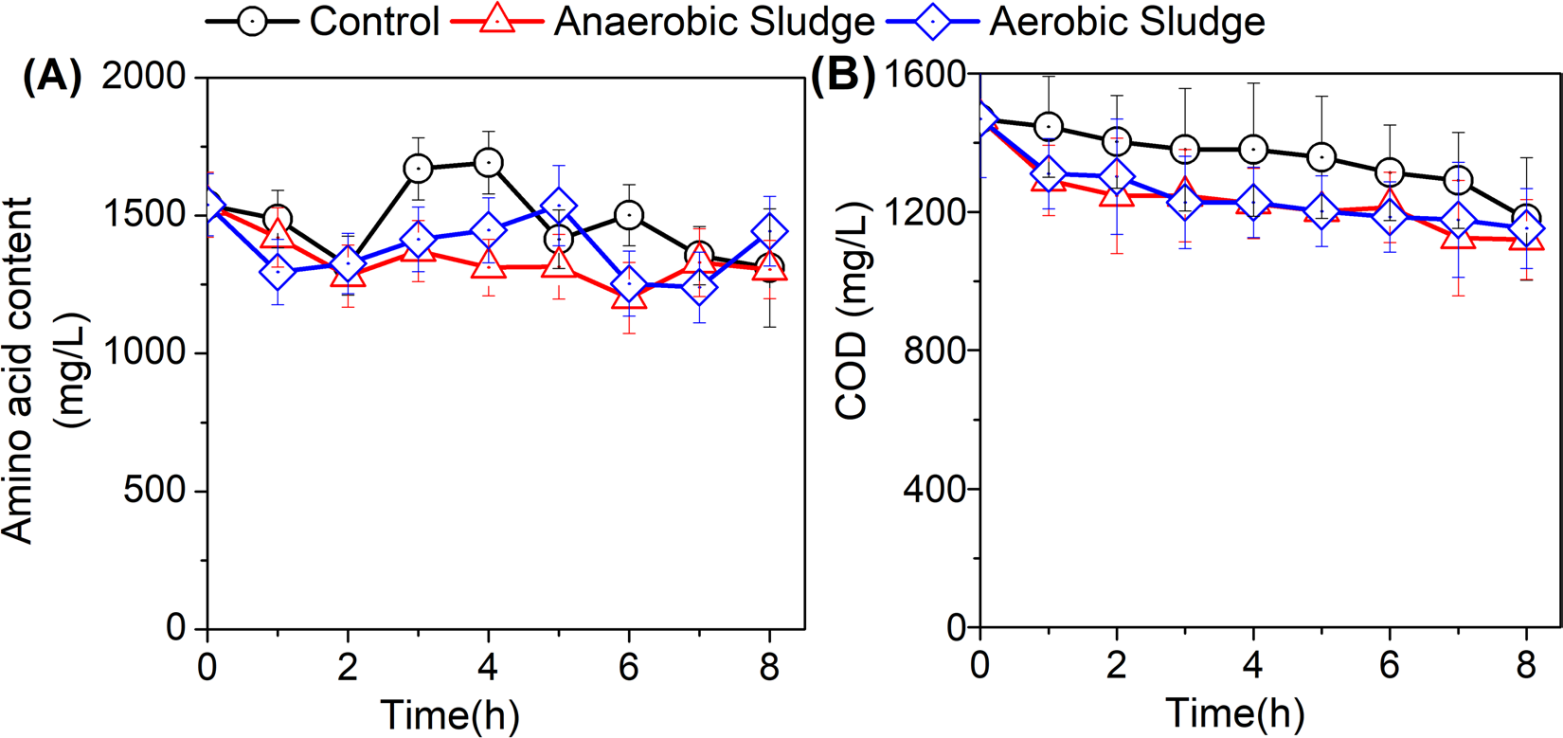
Effects of aeration on AD effluent amino acids (**A**) and COD (**B**) with the addition of activated and anaerobic sludge, respectively.

Amino acids are important nutrients for crop growth and disease resistance^22^. In this study, the content of amino acids varied significantly with aeration (Fig. 2A). In specific, without any sludge addition, the content of amino acids in AD effluent decreased with aeration and then increased the peak of approximately 1692.3 mg/L when aerated for 4 hours, and decreased thereafter. This observation could be attributed to the oxidization of macromolecular substances to peptides, amino acids and other small molecular organic matter. With the addition of activated sludge, the content of amino acids increased to the peak value of 1536.6 mg/L when aerated for 5 hours. By contrast, a continuous decrease in the content of amino acids in AD effluent was observed with the addition of anaerobic sludge. Results from Fig. 2A suggested that aeration to AD effluent resulted in the highest content of amino acids between 3 and 5 hours, where the ration of gas and water was in the range of 20:1–30:1 (i.e. the volume ratio between air and treated AD effluent).

COD is an important physical and chemical index to indicate the total organic contents of AD effluent. As mentioned above, AD effluent contained the main proportion (67.4%) of COD in the digestate (Table 1). In this study, the COD content of all treatments decreased continuously with aeration (Fig. 2B), due to the decomposition of macromolecular organic substances. More significant decrease was observed for the two treatments with sludge additions, indicating the enhanced degradation of organic substances with sludge inoculums.

### Variation of total nitrogen and ammonium nitrogen

**Figure 3:**
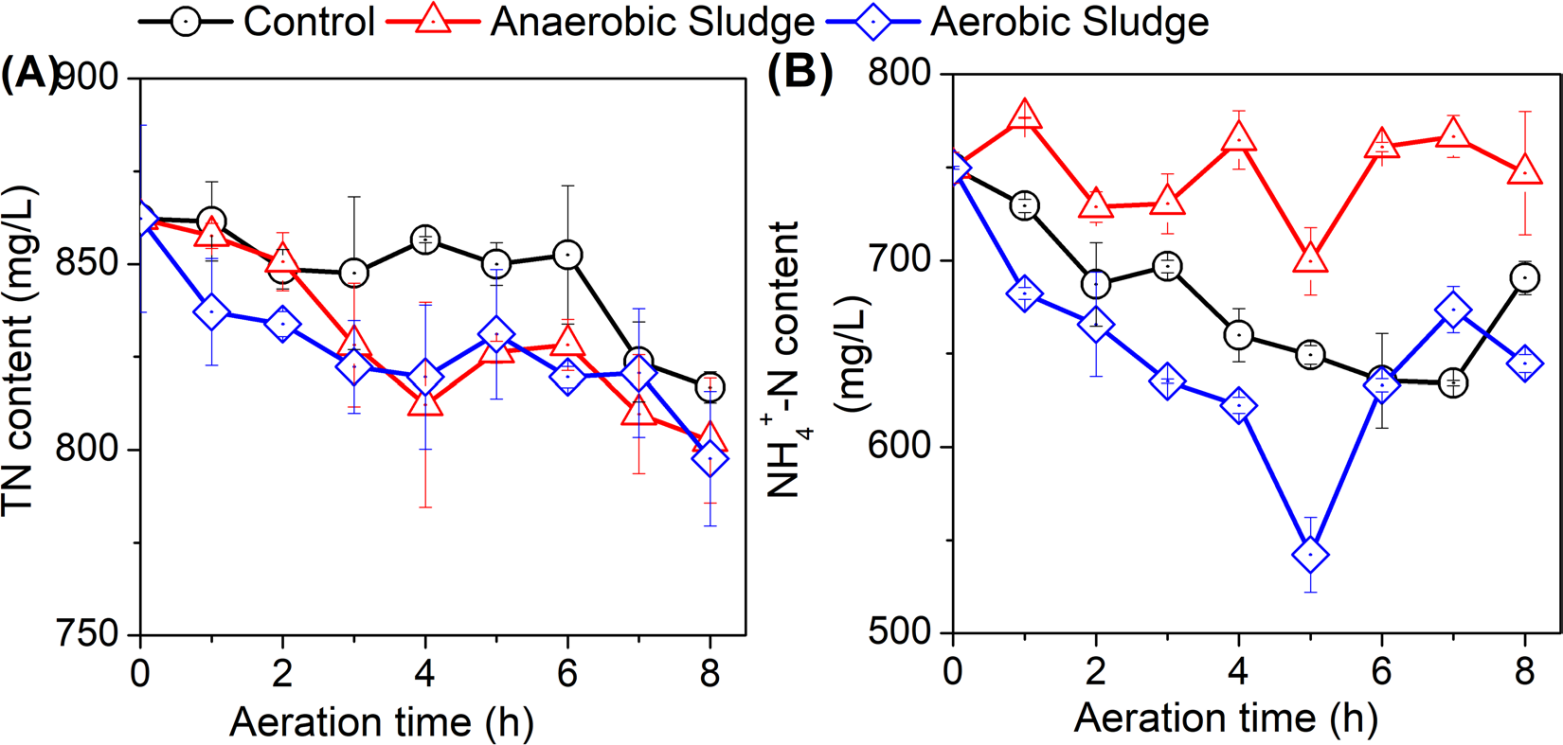
Effects of aeration on the (**A**) TN and (**B**) NH_4_^+^-N in AD effluent with the addition of activated or anaerobic sludge.

Nitrogen is a major nutritional element for plant and crop growth. As shown in Fig. 3A, regardless of sludge addition, the TN content of all treatments decreased gradually with aeration. Such TN reduction could be ascribed to ammonium stripping by aeration^23^. More considerable decrease in TN could be achieved by adding either activated or anaerobic sludge, possibly due to the enhanced nitrogen volatilization and microbial assimilation with sludge addition.

The NH_4_^+^ content in AD effluent varied significantly for the three treatments (Fig. 3B). With the addition of activated sludge, the NH_4_^+^ content decreased considerably and reached the lowest value of 542.2 mg/L after aerated for 5 hours. On the other hand, a more slim decrease was observed for the control treatment without any sludge addition, which attained the valley of 634.2 mg/L when aeration for 7 hours. This deviation was possibly due to introduce of the nitrification process with the addition of activated sludge^24^. Of the three treatments, the highest NH_4_^+^ content in AD effluent occurred when anaerobic sludge was added. This observation could be attributed to the relatively high NH_4_^+^ residual in anaerobic sludge, thereby increasing the NH_4_^+^ content in AD effluent (Fig. 3B).

### Variation of total phosphorus and total potassium

**Figure 4:**
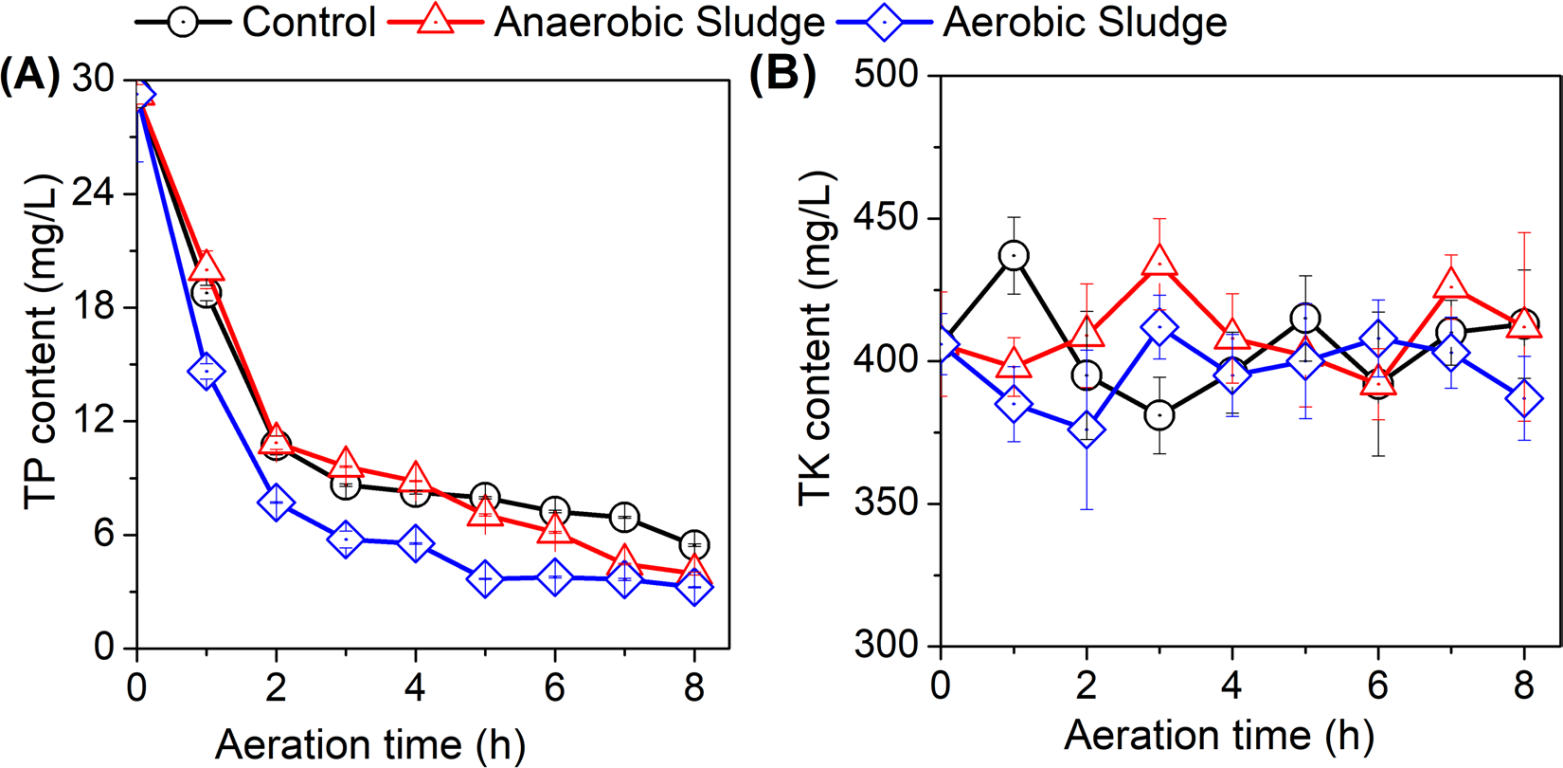
Effects of aeration on the (**A**) total phosphorus (TP) and (**B**) total potassium (TK) in AD effluent with the addition of activated or anaerobic sludge.

As mentioned above, total phosphorus was mainly resided in the solid fraction of digestate with only 47% in the AD effluent. In this study, all treatments experienced a continuous decrease in the TP content in the AD effluent with aeration (Fig. 4A). Given the abiotic condition of the control treatment, such TP reduction could be mainly attributed to the chemical precipitation with an increase in the AD effluent pH. Indeed, a continuous decrease in the magnesium and calcium contents in AD effluent was observed in the following section. Of the three treatments, the treatment with activated sludge addition had the most significant TP decrease. In addition to the chemical precipitation with pH increase, the addition of activated sludge might introduce phosphorus accumulating organisms to enhance microbial assimilation of TP. Unlike the TP content, the total potassium (TK) content in AD effluent was slightly affected by aeration either with or without sludge addition (Fig. 4B).

### Variation of moderate elements

**Figure 5:**
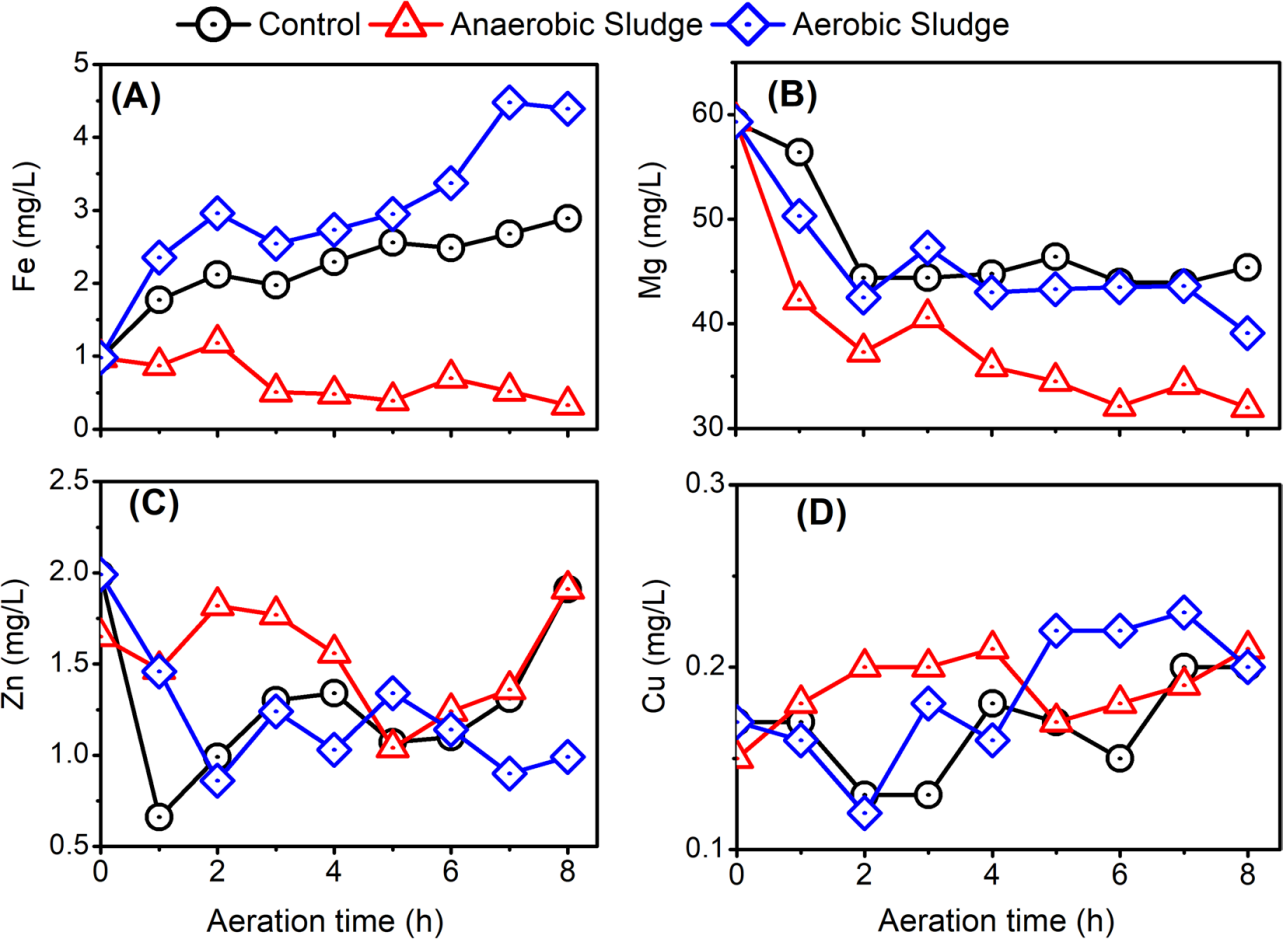
Effects of aeration on the (**A**) Fe, (**B**) Mg, (**C**) Zn, and (**D**) Cu in AD effluent with the addition of activated or anaerobic sludge.

Dominant heavy metals, including Cu, Fe, and Zn, in AD effluent were investigated in this study (Fig. 5). As shown in Fig. 5A, the Fe content in AD effluent increased significantly for the control treatment, and particularly the treatment with the addition of activated sludge. This result could be attributed to the enhanced release of Fe into AD effluent from the activated sludge. Conversely, the Fe content in AD effluent decreased slight when anaerobic sludge were added. The reason for this small decrease was not clear, possibly due to the utilization of Fe by anaerobic sludge since Fe is a key trace element for anaerobic microbes. As discussed above, phosphorus precipitation occurred during the aeration treatment of AD effluent. Thus, magnesium content decreased considerably in all treatments (Fig. 5B). Unlike Fe, despite the notable fluctuation, the contents of Zn and Cu in AD effluent was varied slightly by aeration pretreatment with or without sludge addition (Fig. 5C,D).

### Germination index

**Figure 6:**
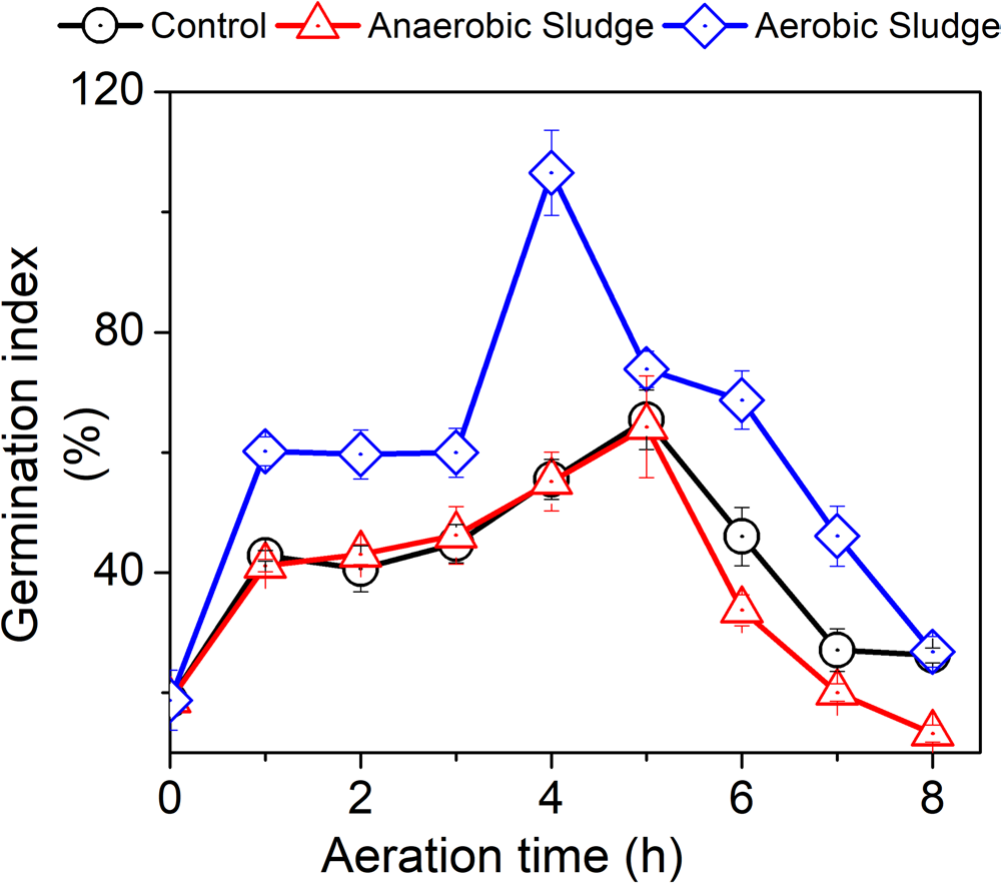
Effects of aeration on the germination index of AD effluent with the addition of activated or anaerobic sludge.

Germination index (GI) was an index to evaluate the biological toxicity of AD effluent to crops. As shown in Fig. 6, the GI value varied significantly for all treatments. Specifically, all treatment exhibited a GI less than 20% before aeration, indicating the unfavourable inhibition of AD effluent to plant growth in agronomic applications. The low GI was also reported by Luo *et al*.^25^ and could be attributed to the high concentrations of NH_4_^+^, sulphate, and volatile organic substances that are resistant to anaerobic digestion^26^. The control treatment and the treatment with the addition of anaerobic digesters experienced a similar GI profile, with the peak of 65.5% and 64.3%, respectively, after aerated for 5 hours.

The GI values of all treatments decreased after aeration for 5 hours (Fig. 6). This observation was possibly due to the oxidization of macromolecular organic substances to small molecular organic acids, which might exert inhibitory effects on crop growth^27,28^. Of the three treatments, the treatment with the addition of activated sludge had the highest GI value throughout the aeration period. The highest GI value of 106.5% could be achieved when aerated for 4 hours. These results suggest that the phytotoxicity of AD effluent could be reduced by aerating for 4–5 hours with the addition of activated sludge. It has been reported that aeration could remove the ammonia nitrogen, phenol, cyanide, sulfide, and other toxic volatile substances from AD effluent, thereby reducing its phytotoxicity^15,29^.

## Conclusion

Results in this study show that aeration with the addition of activated sludge could reduce the phytotoxicity of AD effluent. During the aeration pretreatment of AD effluent, the addition of activated sludge could enhance the AD effluent pH and thus induce chemical precipitation to reduce the AD effluent salinity. Moreover, compared to the control treatment, more considerable decrease in the NH_4_^+^ content was observed with the addition of activated sludge. As a result, a higher GI value was achieved for the treatment with activated sludge indicating a low phytotoxicity to crops, particularly after aerated for 4–5 hours to obtain the ratio of gas and water of 20:1–30:1. By contrast, the addition of anaerobic digesters had negligible effects on the phytotoxicity of AD effluent during its aeration pretreatment. This observation suggests the necessity of liquid-solid separation of digestate to increase the efficiency of aeration pretreatment.

## Material and Methods

### Experimental set-up

**Figure 7:**
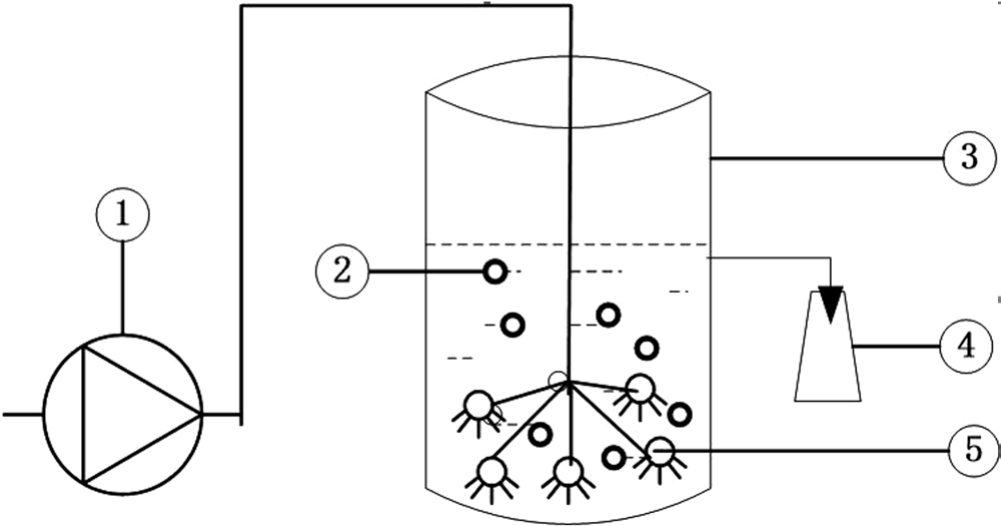
Schematic diagram of aeration system.

The reactor used in the study for AD effluent aeration was a 10 L container (Fig. 7). A microporous aeration device was positioned at the bottom of the reactor. The aeration rate was maintained at 0.036 m^3^/h for sludge acclimatization and AD effluent experiments.

### AD effluent and sludge acclimatization

AD digestate was obtained from a local, large-scale swine farm (Shunyi, Beijing, China), which swine feces were anaerobically digested by an up-flow anaerobic sludge bed (UASB) system. The USAB system had an effective volume of 700 m³. The sludge retention time (SRT) and system temperature was maintained at 12 days and 32 °C, respectively. The USAB system could daily treat 5 t swine feces (both manure and urine) with moisture content of 80% to produce 300 m³ biogas for beneficial use, 6.25 m^3^ AD effluent, and 2 t AD biosolids with moisture content of 65% after centrifugation for liquid and solid separation.

Anaerobic sludge was obtained from the UASB system in the large-scale swine farm (Shunyi, Beijing, China). Activated sludge was collected from a local domestic sewage treatment plant (Haidian, Beijing, China). Both anaerobic sludge and activated sludge were mixed with diluted AD effluent (3 times dilution) to adjust a mixed liquor suspended solid (MLSS) concentration of approximately 4000 mg/L and then aerated intensively for 36 hours in the reactor (Fig. 7). Anaerobic sludge was used to investigate the effect of direct aeration on the nutrient content and phytotoxicity of manure digestate. Activated sludge was inoculated given its high capability for wastewater treatment. Thereafter, the mixed liquor was acclimatized by feeding the diluted and original AD effluent for seven days, respectively. Sludge acclimatization to AD effluent was indicated by the relatively stable specific oxygen up-take rate of approximately 2.7 and 1.2 mg O_2_/g MLVSS (mixed liquor volatile suspended solid) per hour for the mixed liquor inoculated with activated and anaerobic sludge, respectively.

### Aeration experiment

In the aeration experiment, 6 L AD effluent and acclimatized sludge samples were fed to the reactors to achieve a MLSS concentration of 2000 mg/L. This sludge concentration is commonly used for the anaerobic – anoxic – oxic (A^2^O) process in wastewater treatment. AD effluent without any sludge addition was used as a control treatment. All reactors were continuously aerated for 8 hours with an aeration rate of 0.036 m^3^/h to provide a dissolved oxygen of approximately 2 mg/L, which is widely used for activated sludge treatment in practice^30^. Mixed liquor samples were collected every hour to measure the nutrient content and biological toxicity of supernatant after centrifugation at 3750 g for 20 minutes. The gas/water ratio, which defined the ratio between the total volume of air provided by aeration over a certain period and the volume of treated AD effluent, was calculated to quantify the efficiency of aeration to mitigate the phytotoxicity of AD effluent with inoculums. All treatments in this study were conducted in triplicate.

### Analytical methods

TN and TP were determined by the alkaline potassium persulfate digestion-UV spectrophotometric method and the ammonium molybdate spectrophotometric method, respectively. NH_4_^+^ was analyzed using a flow injection analysis system (QuikChem 8500, Lachat, CO). COD was measured by the fast digestion spectrophotometric method with high range COD vials (HACH, USA). Heavy metals and salt cations were analyzed using the inductively coupled plasma- optical emission spectrometry. Amino acids were analyzed based on the Chinese Standard Method of Liquid Fertilizer (NY/T1975-2010). EC and pH were determined by using a pH/conductivity meter (Orion 4-Star Plus, Thermo Scientific, Waltham, MA). AD effluent samples before and after aeration experiment with sludge addition were diluted for 5 times and then filtered through a 0.45 μm glass fiber filters to determine the seed GI based on the method described previously by Yang *et al*.^31^. *Pakchoi* seeds were used in this study.

Mean values and standard deviations of triplicate experiments were shown in this study. The SAS System for Windows V8.0 was used for the variance analysis.

## Data Availability

The authors declared that none of the data in the paper had been published or was under consideration for publication elsewhere.
